# Perception and utilisation of veterinary services by rodent owners in the United Kingdom

**DOI:** 10.1002/vetr.4958

**Published:** 2025-01-25

**Authors:** Lucy James, Alison P. Wills

**Affiliations:** ^1^ Department of Animal and Agriculture Hartpury University Gloucester UK

## Abstract

**Background:**

There is limited research on how rodent owners use and perceive veterinary services and what the demand for pet insurance for these species is.

**Methods:**

An online survey of owners of pet rodents (guinea pigs, hamsters, rats, gerbils and mice) measured owner confidence in recognising signs of illness, their opinions on and use of veterinary services and their willingness to purchase pet insurance.

**Results:**

A total of 1700 respondents completed the survey. Rat owners had increased confidence in recognising signs of illness, as did owners who acquired their pet from a breeder or rescue centre. Most respondents had used veterinary services, with owners perceiving exotics specialists to have increased knowledge. Economic challenges with accessing veterinary care were common. Where rodents were purchased for children, the amount owners were willing to spend on veterinary care was significantly less.

**Limitations:**

Responses may have been biased towards keen owners who self‐selected to participate in the survey. Respondents were predominantly female, which may have affected willingness to access and pay for veterinary services.

**Conclusion:**

Veterinary spending was not affected by income; however, owners who purchased their animal for their children were less willing to pay for veterinary services and pet insurance.

## INTRODUCTION

Rodents have historically been regarded as disposable pets^1^; however, expectations for high‐quality veterinary care have increased alongside ownership numbers.^2^ Rodents are popular pets, with UK ownership figures in 2023 as follows: guinea pigs (1,000,000), hamsters (900,000), rats (600,000), gerbils (500,000) and mice (200,000).^3^ While rodents are well studied as laboratory species, disease prevalence in pet rodents is comparatively poorly documented.^4^ Diagnosis of diseases in rodents may be challenging for veterinarians due to reduced clinical exposure to these species and a paucity of information on their common disorders.^5^ Additionally, rodents are evolutionarily adept at masking signs of illness, and smaller species have a reputation for being difficult to handle.^6^ Some recent studies have documented the common diseases of guinea pigs^7^, ^8^ and hamsters,^5^ but peer‐reviewed literature remains limited. Research has also examined the husbandry of pet guinea pigs in the UK, identifying issues with housing and the provision of the correct diet and companionship.^9^ Research has demonstrated that demographic factors including gender and earnings affect the housing provided to pet rabbits.^10^ In addition, rabbits purchased for children as a ‘starter pet’ were less likely to receive regular healthcare, and owners were less willing to pay for veterinary treatment.^11^ Similarly, owners with children and those with a dog or cat had a weaker bond with their rabbits.^12^ It is unknown whether the same applies to pet rodents, as these species are also commonly purchased for children,^13^ and whether the reason for purchase affects owners' willingness to pay for veterinary services.

Rodents are usually meticulous groomers and can remove external evidence of underlying illness,^14^ so regular health checking by owners is important to detect minor changes. Additionally, interactions between conspecifics may affect feeding behaviours.^15^ Anorexia is a common sign of illness,^16^ and enclosure sharing can make it difficult for owners to monitor the feed intake of each pet. Interestingly, research has shown that more guinea pig owners would seek rapid treatment for skin parasites than for serious presentations like anorexia.^9^ This suggests that owners may lack the ability to prioritise the severity of clinical signs, meaning animals may be presented to the veterinarian in a more debilitated state.

To date, there is limited research on how rodent owners access and perceive veterinary services. Research investigating the practices of rat owners identified that 79% had used veterinary services, but a lack of species‐specific knowledge among veterinarians was reported.^17^ This could be due to a low frequency of rodents being presented, which would decrease the opportunity for experiential learning,^18^ or limited training with exotics that may impact veterinarians' knowledge and confidence when treating them.^19^ A survey‐based study of guinea pig owners found that 74.4% took their guinea pig to a veterinarian if they believed they were unwell^9^; however, one study reported that only 25.8% of hamsters had been presented to a veterinarian.^20^ This could be a result of good health in hamsters; however, as prey species, they may be hiding signs of illness. Owners may also be reluctant to spend money on rodents due to their short lifespan, or they may be perceived as a ‘cheap’ pet^4^ with veterinary care deemed unnecessary. A lack of routine vaccinations or microchipping may also mean fewer rodents are presented to veterinarians in comparison to dogs, cats and rabbits, reducing the likelihood of diseases being diagnosed at an early stage.^4^, ^17^

Pet insurance assists in the cost of diagnostic testing and treatment for unforeseeable health conditions, where the insurance company reimburses the owner following a claim submission.^21^ Since 2021, the UK cost of living crisis has impacted veterinary practices, with 56% of veterinarians stating that clients cannot afford sporadic veterinary bills, and 9% of owners delayed presenting their ill pet to a veterinarian due to inadequate funds.^22^ Advanced veterinary care is associated with considerable expense, and optimal outcomes may be restricted by a client's financial situation. Pet insurance may alleviate these challenges by increasing the ability to undertake necessary treatment interventions.^23^ In the UK in 2019, 57% of dogs and 37% of cats were insured, with approximately 80 companies offering pet insurance.^23^ Meanwhile, in the UK in 2014, 11.7% of pet rabbits were insured,^24^ but it is likely that fewer rodents were insured, and more recent data are needed. Rodent owners’ willingness to purchase insurance may be limited by the lack of companies offering insurance plans and the lifespan of these species. To the authors’ knowledge, there have been no previous publications directly examining the demand for exotic pet insurance in the UK.

The aim of the present study was to ascertain the extent to which rodent owners in the UK use veterinary services and their perceptions of the quality of services offered.

## METHODS

An online survey was created and was open from 1 to 25 November 2023. Social media platforms, such as Facebook and LinkedIn, were used to recruit participants. The survey was also shared on the social media accounts of a UK‐based lay press publication, ‘Guinea Pig Magazine’. Convenience and snowball sampling were employed through sharing the survey in rodent‐specific Facebook groups, as well as on the first author's (LJ) personal platform and non‐pet groups to help mitigate the participation bias seen in rodent groups. This study was approved by the Hartpury University Ethics Committee (ETHICS2023‐14).

### Survey design

An online quantitative survey consisting of 22 questions was created on Microsoft Forms to assess rodent owners' opinions of veterinary services and their confidence in recognising illness. Respondents were asked to provide demographic data including their gender, age and yearly income. Data were also collected on what species of rodent the respondent owned, if they also owned a cat and/or dog, where they acquired the rodent and whether it was purchased for a child.

Respondents were asked to provide their opinion on and prior experience of various aspects of veterinary care and services, including whether they had presented their pet to a vet (including an exotics specialist), their confidence in the veterinarian and their willingness to pay for veterinary care and/or pet insurance. Questions also explored why respondents had or had not chosen to purchase insurance for their pet, and where cats and/or dogs were also owned, whether these species were insured. Respondents were also asked their opinion on the cost of veterinary services, including medication and consultation costs. They were also asked to rate the strength of their bond with their rodent.

In addition, respondents were asked to rate their agreement with the statement ‘I am confident in assessing when my pet is ill’, and they were also presented with a range of clinical signs and behaviours and asked whether these were signs of illness (yes or no options). The number of correct responses was summed to give each respondent a score out of nine for this question.

The inclusion criteria for respondents required them to be UK residents, aged 18 or above, who currently owned a rodent (guinea pig, hamster, rat, gerbil or mouse). If respondents owned multiple animals and/or species, they were asked to respond for the pet they had owned the longest. Participants were given the opportunity to withdraw their responses up to the point of data processing by contacting the researcher and quoting a six‐digit identifier that they created when completing the survey.

Closed‐ended and five‐point Likert‐scale questions were used within the survey. Some Likert‐scale questions had an extra category of ‘non‐applicable’ where appropriate. One open‐ended question was used to allow respondents who had seen an exotics specialist to elaborate on this experience.

### Data analysis

All statistical analyses were performed in IBM SPSS Version 29. Likert scales were coded with ‘strongly agree’ = 1 through to ‘strongly disagree’ = 5. Respondents were given an overall score for their responses to the questions about signs of illness in rodents. Data were considered non‐parametric due to the ordinal and nominal nature of the questions, and thus the median and range were reported. Spearman's rank correlation was used to identify if there was a correlation between respondents’ reported confidence in identifying signs of ill health and their score for recognising these signs. A chi‐squared test was applied to determine if demographic variables, such as age, were associated with willingness to buy pet insurance. Mann–Whitney *U* and Kruskal–Wallis tests were used to identify significant differences between demographic groups regarding their opinions of veterinary services. A Kruskal–Wallis test was used to assess for differences between species owned in the score attributed to respondents for correct identification of signs of ill health. Where significance was identified in the Kruskal–Wallis test, Dunn's post hoc tests with Bonferroni correction were applied to identify significant differences between groups. The level of significance was set at *p* < 0.05.

## RESULTS

### Respondent demographics

A total of 1710 surveys were completed but 10 were excluded due to respondents residing outside of the UK, resulting in 1700 surveys being analysed. These provided information from the owners of 775 guinea pigs, 500 hamsters, 288 rats, 87 gerbils and 50 mice. Most respondents identified as female (95%; *n* = 1614), had a median age of 25–34 years and earned a median annual income of £20,001–£30,000. There were respondents from all regions in the UK, with most responses gained from the south west (13.9%; *n* = 236). A pet shop was the most common location for obtaining rodents (37%; *n* = 644), with 17% (*n* = 296) being purchased for children. In addition to their rodents, 58% (*n* = 975) owned a dog or a cat.

### Confidence in recognising ill health

There was a statistically significant association between respondent age and confidence in recognising ill health in pet rodents (*H*(5) = 16.756, *p* = 0.005). However, pairwise comparisons showed no significant differences between individual groups. Income (*H*(5) = 27.007, *p* < 0.001), species (*H*(4) = 22.205, *p* < 0.001) and where the pet was obtained (*H*(4) = 32.208, *p* < 0.001) were also significantly associated with confidence in recognising ill health. People with an income of between £40,001 and £50,000 were less confident in assessing whether their pet was ill than those who earned below £10,000 (*p* = 0.039), between £10,001 and £20,000 (*p* = 0.020) or between £20,001 and £30,000 (*p* = 0.007). In addition, those who earned between £20,001 and £30,000 were more confident than those who earned between £30,001 and £40,000 (*p* = 0.040) and those who earned above £50,000 (*p* = 0.021). Rat owners had the highest confidence in assessing when their pet was ill, higher than guinea pig owners (*p* = 0.001) and hamster owners (*p* < 0.001). People who obtained their pet from a rescue centre (*p* = 0.002) or breeder (*p* < 0.001) were more confident in assessing when their pet was ill than those who got their pet from a pet shop. In addition, those who were gifted a pet were less confident than those who obtained their pet from a breeder (*p* = 0.002) or rescue centre (*p* = 0.003). All other pairwise comparisons were non‐significant (*p* > 0.05) and can be seen in Supporting Information S1.

There was a significant association (H(4)= 51.615, *p* <0.001) between the species owned and owner knowledge of clinical signs of ill health. When asked to identify clinical signs indicating poor health, rat owners scored significantly higher (*p* <0.001) than guinea pig, gerbil and mouse owners. There was a weak positive correlation (*r* = 0.128) between perceived confidence in recognising ill health and score when recognising clinical signs, with higher levels of perceived confidence resulting in higher scores.

### The pet–owner bond

The pet‐owner bond was weaker for owners who purchased their rodent for their children (*U* = 153,796.50, *p* < 0.001).

### Perceptions of different types of veterinary services

Most respondents had presented their rodent to a veterinarian (77%, *n* = 1320), and 43% (*n* = 571) had previously been referred to a specialist in exotic species or were already registered with one. Generally, owners who had visited a veterinarian (*n* = 1320) were satisfied with the veterinarian's diagnosis (66%, *n* = 871). Owner rating of the knowledge of their own veterinarian was significantly higher (U = 285,848.500, *p* <0.01) for those who had seen a exotics specialist than for those who had only presented their rodent to a general practice veterinarian. Owners were also more confident with the diagnosis of an exotic specialist veterinarian compared to a general practice veterinarian (*U* = 270,344.500, *p* < 0.001). Respondents who had been referred to a specialist in exotics (*n* = 183) provided insight into their opinions (Table 1). Abundance of knowledge and confidence were common themes, in addition to the expense of seeing specialist veterinarians. However, the wider variety of medications offered by exotic specialists was appreciated by owners. Some respondents stated that specialist veterinarians were extremely compassionate, while others described the lack of compassion as the main negative aspect of their experience.

**TABLE 1:** Common themes identified from responses to the question ‘Could you briefly describe your experiences with a specialist exotics vet’ (*n* = 183), presented in descending order of the number of responses that mentioned these areas

**Common themes**	**Number of responses**
Confident/knowledgeable	77
Expensive	30
Negative emotional experience	12
Compassion (positive)	7
Compassion (negative)	6
Alternative medication offered	5

### Perceptions of cost and willingness to pay for veterinary services

Opinions regarding the affordability of medication significantly varied between age groups (*H*(5) = 11.323, *p* = 0.045) and with species owned (*H*(4) = 10.866, *p* = 0.028). Other demographic variables, such as income, did not significantly affect willingness to pay for veterinary services (*p* > 0.05). There was a statistically significant association between age (*H*(5) = 35.105, *p* < 0.001), species (*H*(4) = 18.436, *p* < 0.001) and where the pet was obtained (*H*(4) = 25.719, *p* < 0.001) and willingness to pay for medication. There was a significant difference between guinea pig and hamster owners with hamster owners finding the cost of medication more reasonable (*p* = 0.013). Respondents who were 18–24 years old were significantly more willing to pay for medication for their pet than those who were 25–34 (*p* = 0.037), 35–44 (*p* < 0.001), 45–54 (*p* < 0.001) and 55–64 (*p* = 0.020). Those who were 25–34 years old were also significantly more willing to pay than those who were 45–54 (*p* = 0.024). Rat owners were significantly more willing to pay for medication than guinea pig (*p* = 0.001) or hamster owners (*p* = 0.007). People who obtained their pet from a rescue centre (*p* < 0.001), breeder (*p* = 0.009) or from another source (*p* = 0.007) were significantly more willing to pay for medication than those who bought their pet from a pet shop. All other pairwise comparisons were non‐significant (*p* > 0.05) and are presented in Supporting Information S2.

A significant association was identified between willingness to pay for medication and whether the rodent was purchased for a child (*U* = 153,842.00, *p* < 0.001), with owners who purchased for a child being less willing than those who did not. Those who purchased a pet for a child were significantly less willing (*U* = 11,889.50, *p* < 0.001) to present the rodent to a veterinarian than those who did not purchase the pet for a child.

### Perceptions of and willingness to purchase pet insurance

Most owners (57%, *n* = 971) reported that they would not be willing to purchase pet insurance for their rodent, 38% (*n* = 653) reported they would purchase insurance and 4% (*n* = 76) already had insurance. The main reason in favour of insurance was financial protection (34.4%), with the main disadvantage being additional monthly costs (33.1%). Most rodent owners who also had a dog or cat (58%, *n* = 975) had pet insurance for their dog or cat (69%, *n* = 682). There was a statistically significant association between both region (*H*(10) = 19.742, *p* = 0.032) and income (*H*(5) = 11.197, *p* = 0.048) and owner perception of the importance of insuring dogs and cats over rodents. However, pairwise comparisons found no significant differences between individual groups (*p* > 0.05). There was a statistically significant association between age group and willingness to purchase insurance (*χ* ^2^(10) = 19.612, *p* = 0.03), with significantly more 18–24‐year olds willing to purchase pet insurance and significantly less 35–44‐year olds willing to purchase insurance. Other demographic variables, such as income, did not significantly affect willingness to pay for pet insurance (*p* > 0.05).

There was also a statistically significant association between species owned and willingness to purchase insurance (*χ* ^2^(8) = 24.832, *p* = 0.002) and where the pet was obtained and willingness to purchase insurance (*χ* ^2^(8) = 27.172, *p* < 0.001; Tables 2 and 3). There was a significant association between having a dog or cat insured and having a rodent insured (*χ* ^2^(2) = 57.691, *p* < 0.001). Significantly more owners who had insured their dog or cat had also insured their rodent. There was a significant association between whether the rodent was purchased for a child and willingness to purchase insurance (*χ* ^2^(2) = 11.421, *p* = 0.003), with respondents who purchased the rodent for a child less likely to purchase pet insurance.

**TABLE 2:** Crosstabulation of species and responses to the question ‘Would you be willing to purchase pet insurance?’

	Would you be willing to purchase pet insurance?		
Species	Yes	No	Already have
Guinea pig	311 (1.3)	418 **(−2.4)**	46 **(2.7)**
Hamster	202 (1.1)	279 (−0.7)	19 (−0.9)
Rat	84 **(−3.5)**	198 **(4.4)**	6 **(−2.2)**
Gerbil	36 (0.6)	47 (0.6)	4 (0.1)
Mouse	20 (0.2)	29 (0.1)	1 (0.9)

*Note*: Adjusted residuals appear in parenthesis below observed frequencies. Significant adjusted residuals are presented in bold text.

**TABLE 3:** Crosstabulation of where the pet was obtained and responses to the question ‘Would you be willing to purchase pet insurance?’

Where pet was obtained	Would you be willing to purchase pet insurance?		
	**Yes**	**No**	**Already have**
Pet shop	284 **(3.8)**	330 **(−3.8)**	30 (0.3)
Rescue centre	149 **(−2.1)**	261 (1.3)	26 (1.7)
Breeder	135 **(−2.1)**	251 **(2.8)**	11 (−1.9)
Gifted	11 (−0.7)	19 (−0.1)	4 **(2.1)**
Other	74 (0.2)	110 (0.3)	5 (−1.3)

*Note*: Adjusted residuals appear in parenthesis below observed frequencies. Significant adjusted residuals are presented in bold text.

## DISCUSSION

Owners were generally confident in recognising clinical signs of illness, with rat owners demonstrating the highest confidence. Increased knowledge and confidence were reported by owners who presented their rodents to veterinarians who were specialists in exotics. Financial limitations associated with veterinary care were common, affecting willingness to purchase medication and pet insurance. Lastly, owners who purchased the pet for a child were less willing to partake in veterinary spending.

### Demographics

Most respondents identified as female, which is consistent with many questionnaire‐based studies.^10^, ^25^, ^26^ Women with no children often develop stronger pet–owner bonds and are more likely to own pets, which could provide motivation to fill out a survey regarding their care.^27^, ^28^ Respondents had a median age of 25–34, with the least engagement from those aged 65 and above. Rodents were most commonly obtained from a pet shop, which is consistent with other studies of pet rodents.^9^, ^29^ This could be due to the convenience of being able to purchase both the pet and resources simultaneously or to enable prospective owners to seek advice.^30^ Despite pet ownership being more likely in families with children,^28^ just 17% of the rodents in the present study were purchased for children; however, in previous literature, 27%–39% of rabbits were purchased for children.^24^, ^26^ This discrepancy may be due to species differences, with rabbits being a more popular pet for children, but literature on how many rodents are purchased for children is currently lacking.

### Confidence in recognising signs of ill health

Owners were generally confident recognising signs of ill health, with a positive correlation between perceived confidence and clinical sign recognition score, which aligns with previous research involving guinea pig owners.^31^ Rat owners had the highest confidence in identifying signs of ill health, perhaps due to a stronger pet–owner bond.^17^ Additionally, rats are a larger and slower species than some of the smaller rodents^2^; therefore, handling is easier, which may result in more efficient health checking. Hamster owners were least confident in identifying signs of ill health in their pets. Hamsters are nocturnal in captivity,^32^ while rats are polyphasic sleepers,^33^ which may affect the frequency of owner interaction and behaviour observation.

Acquiring a pet from a rescue centre or breeder was associated with higher confidence in identifying signs of ill health than purchasing from pet shops or being gifted the rodent. This is consistent with research that demonstrated that rabbit owners who acquired their pet from a rescue centre had the greatest health and welfare knowledge.^34^ While more research is needed to investigate this association, those who are more likely to rescue may already possess enhanced knowledge on their chosen species, whereas those who were gifted a pet may have reduced confidence due to having limited time to complete prior research.^35^ Owners who purchased the rodent for a child had reduced confidence in recognising illness in their pet. It may be that these pets were more likely impulse purchases, dependent on the child's interest or because of media trends.^36^ People with lower incomes were more confident in identifying signs of ill health. The reasons for this are unclear, but these could be younger people who may be purchasing the pet for themselves rather than for a child. These individuals may therefore be more invested in their pet and likely to identify signs of ill health.

### Perceptions of veterinary services

As rodents are popular pets, it is important for veterinarians to be knowledgeable regarding their health and husbandry.^37^ In the present study, most rodents were purchased for an adult and had been presented to a veterinarian. Furthermore, owners who had used exotic specialist veterinarians (either as first opinion or following referral) had greater confidence in their veterinarian's expertise than respondents who had only used general practice veterinarians. While this finding supports the use of specialist veterinarians for rodents, this may not be possible for all owners due to the limited accessibility of these veterinarians and the increased cost involved. It has already been highlighted in the literature that affordability of veterinary care may be challenging for some owners due to the range of cost‐intensive diagnostic and therapeutic options,^38^ limiting the quality of care a veterinarian can provide, which can cause frustration for both owners and veterinarians.^39^ The present study found that owner age was associated with willingness to pay for medication, with significant differences between younger adults and middle‐aged adults. Despite middle‐aged adults having the highest average disposable income,^40^ their reduced willingness to pay may be a result of alternative spending priorities, especially if they have dependents. This may also account for the lower willingness to present their rodent to a veterinarian and lower willingness to pay for medication from owners who purchased the rodent for a child. Interestingly, annual income was not associated with willingness to pay for veterinary services in the present study.

The veterinarian–client bond affects the perception of care quality and value,^41^ yet there were mixed reports of the compassion offered by exotic specialist veterinarians in the present study. Often, there is not a pre‐existing rapport between the client and a referral veterinarian, which may affect the compassion shown due to limited knowledge of the client's history.^42^ Subsequently, reduced trust in their ability to offer compassionate care may present a barrier to seeking veterinary attention.^43^ Successful treatment recommendations should be supported by empathy and trust; however, confusion and poor veterinary support are more likely to result in a negative experience.^41^ Rodents can deteriorate rapidly when unwell, and therefore may be presented in a more severe state, causing a worse prognosis^9^ and subsequent emotional distress in owners. Veterinarians often recommend twice‐yearly veterinary check‐ups to recognise early indicators of disease, allowing rapid therapeutic intervention. However, previous studies found that only 22% of rabbits and 12.8% of guinea pigs receive regular health checks,^9^, ^11^ with the frequency for other rodent species likely lower.

Despite rodents becoming more commonly presented in veterinary practices, there are limited licensed therapeutics available for their use,^44^ which may impact owner willingness to pay for medication. However, owners who used a specialist in exotic species appreciated the wider variety of medications available.

### Pet insurance

While many owners were not willing to purchase pet insurance for their rodents, there is still some demand for insurance. Although this demand is lower than in other companion animals such as dogs, where 65% of owners would be willing to purchase insurance,^38^ there has been an incline in interest for other species.^45^ In the present study, 4% of rodents were insured. This is a low number relative to ownership popularity, and a lot less than for dogs, cats and rabbits, where 27.9%, 18.5% and 9.1% were insured, respectively.^46^ This may be a result of reluctance to spend money on animals that are commonly perceived as a ‘cheap pet’.^4^ Furthermore, companies offering insurance for rodents are much more limited than for dogs, cats and rabbits, meaning owners may not be aware of the options available or may lack confidence in less well‐known insurance companies. Previous research has also shown that 45% of owners would rather use savings to pay for sporadic vet bills, which may also be the case for rodent owners.^38^ Hidden snags in insurance policies may also deter owners from insuring their pets.^47^

Rodent owners aged 18–24 were more likely to purchase pet insurance, which aligns with research into dog owners where the likelihood of purchasing pet insurance was higher for younger owners.^45^ The present study found that, for respondents who owned multiple species, having a cat or dog insured was significantly associated with having a rodent insured. In previous research, dog and cat owners had a weaker bond with their pet rabbits,^12^ which may cause owners to prioritise their spending on dogs and cats^47^ rather than rodents. Guinea pig owners were most likely to already have insurance, whereas rat owners were less likely to purchase insurance. Guinea pigs have the longest lifespan of the rodent species included in the present study, living an average of 5–7 years,^48^ whereas rats only live for approximately 2 years,^17^ which may influence the decision not to insure. Conversely, rat owners were more willing to purchase medication for their pet than guinea pig and hamster owners, potentially as a result of a stronger pet–owner bond.

Acquiring a rodent from a pet shop was associated with reduced willingness to pay for medication, but was associated with increased willingness to purchase insurance. Owners who acquired their pet from a breeder or rescue centre were less likely to get their pet insured. The reasons for this are unclear; however, rescued pets may have pre‐existing medical conditions,^49^ which may deter owners from taking out insurance if these conditions cannot be covered. Purchasing a pet for a child was associated with reduced willingness to purchase insurance. This could be a result of the perception that these species are a ‘starter pet’, which has been found to significantly lower the chance of providing regular healthcare in rabbit owners who purchased the pet for a child.^11^ Previous research also showed that owners with children had a weaker bond with their pets, which ultimately affects the perceived value of the animal, possibly consequently affecting consumer spending.^50^

### Limitations and future research

The main limitation of this study was that enthusiastic, knowledgeable owners may have been overrepresented in the sample population, which is common for pet‐based questionnaire studies.^9^, ^10^, ^20^ However, as many of these respondents had used veterinary services, the data are still meaningful. This limitation could be addressed in future work by conducting a nationally representative study of rodent owners. Further study regarding owners' opinions of veterinary services for rodents using a qualitative approach could allow for deeper understanding of the experiences and reasoning of participants.^51^ Future work could also use focus groups to explore the opinions of pet owners and veterinarians on the care of rodents.^52^ As the present study asked respondents to complete the survey for one pet, further research could consider how the number of pets owned affects owner spending on veterinary care, as some rodent species require conspecific company and the number of animals owned may alter spending behaviour. Similarly, the research conducted by owners before acquiring their rodents could be investigated in relation to veterinary service use, as those who impulse purchased their pets may show a different frequency of presentation compared to experienced owners, as seen in rabbit owners.^26^ In the present study, owners self‐rated their knowledge of signs of ill health in their pets; however, it is possible they did not accurately rate their own knowledge. Future work could further compare owner‐reported awareness of the health and husbandry of rodents to their actual knowledge. In this study, the survey asked about experiences with veterinarians who specialise in exotics, but participants were not asked what qualifications, if any, specialists they had seen had held. This is something that could be explored in further work to distinguish between veterinarians holding specialist qualifications and those who just have an interest and expertise in rodents.

In conclusion, owners generally perceived themselves to be knowledgeable on clinical signs of ill health in their pets. Owners' perception of their veterinarian's knowledge was higher for those who had presented their pet to exotic specialist veterinarians. Annual income did not affect willingness to pay for veterinary services in the present study. However, where rodents were purchased for children, owners were less confident in identifying signs of illness, less willing to spend money on veterinary care and less likely to purchase pet insurance. This does present a concern about the welfare of rodents purchased as pets for children, and more work is required to explore this issue further.

## AUTHOR CONTRIBUTIONS

Lucy James and Alison Wills both contributed to study design. Lucy James administered the survey and analysed the data. Lucy James and Alison Wills both contributed to the manuscript creation.

## CONFLICT OF INTEREST STATEMENT

The authors declare they have no conflicts of interest.

## ETHICS STATEMENT

Ethical approval for this study was granted by Hartpury University Ethics Committee on 27 October 2023 (ETHICS2023‐14).

## FUNDING INFORMATION

This project did not receive any funding from any agency in the public, commercial or not‐for‐profit sectors.

## Supporting information



Supporting Information

Supporting Information

Supporting Information

## Data Availability

The data that support the findings of this study are available from the corresponding author upon reasonable request.

## References

[1] Tamura Y . Current approach to rodents as patients. J Exot Pet Med. 2010;19:36–55. 32288672 10.1053/j.jepm.2010.01.014 PMC7106322

[2] Keeble E . Guide to veterinary care of small rodents. In Pract. 2021;43:424–437.

[3] UK Pet Food . Historical pet data. UK Pet Food; 2023. Accessed April 23, 2024. https://www.ukpetfood.org/industry‐information/statistics‐new/historical‐pet‐data.html

[4] Meredith A . Guinea pigs: common things are common. Vet Rec. 2015;177:198–199. 28319923 10.1136/vr.h4465

[5] O'Neill DG , Kim K , Brodbelt DC , Church DB , Pegram C , Baldrey V . Demography, disorders and mortality of pet hamsters under primary veterinary care in the United Kingdom in 2016. J Small Anim Pract. 2022;63:747–755. 35732354 10.1111/jsap.13527 PMC9796486

[6] Pellett S , Mancinelli E . Veterinary care of hamsters. Part 2: diagnostics, diseases. Companion Anim. 2017;22:743–749.

[7] O'Neill DG , Taffinder JL , Brodbelt DC , Baldrey V . Demography, commonly diagnosed disorders and mortality of guinea pigs under primary veterinary care in the UK in 2019—a VetCompass study. PLoS One. 2024;19:e0299464. 38536813 10.1371/journal.pone.0299464 PMC10971687

[8] Minarikova A , Hauptman K , Jeklova E , Knotek Z , Jekl V . Diseases in pet guinea pigs: a retrospective study in 1000 animals. Vet Rec. 2015;177:200. 26198213 10.1136/vr.103053

[9] Harrup A , Rooney NJ . Current welfare state of pet guinea pigs in the UK. Vet Rec. 2020; 186:282. 32054719 10.1136/vr.105632

[10] Mee G , Tipton E , Oxley JA , Westgarth C . Owner demographic factors are associated with suitable pet rabbit housing provision in the United Kingdom. Vet Rec. 2022;190:e1736. 35661365 10.1002/vetr.1736

[11] Skovlund CR , Forkman B , Lund TB , Mistry BG , Nielsen SS , Sandøe P . Perceptions of the rabbit as a low investment ‘starter pet’ lead to negative impacts on its welfare: results of two Danish surveys. Anim Welf. 2023;32:e45. 38487438 10.1017/awf.2023.41 PMC10936283

[12] Přibylová L , Součková M , Kolářová MF , Vostrá‐Vydrová H , Chaloupková H . Does a stronger bond with pet rabbits equate to better husbandry conditions for them? Appl Anim Behav Sci. 2024;270:106143.

[13] Kottferová L , Molnár L , Čonková E , Major P , Sesztáková E , Szarková A , et al. Fungal flora in asymptomatic pet guinea pigs and rabbits. Animals. 2022;12:2387. 36139247 10.3390/ani12182387 PMC9495200

[14] Frohlich J . Rats and mice. In: Quesenberry K , Mans C , Orcutt C . Ferrets, Rabbits and Rodents. Elsevier Health Sciences; 2020. p. 345–367.

[15] Ali MA , Kravitz AV . Challenges in quantifying food intake in rodents. Brain Res. 2018;1693:188–189. 29903621 10.1016/j.brainres.2018.02.040 PMC6005200

[16] Quesenberry KE , Donnelly TM . Providing a home for a guinea pig. MSD Veterinary Manual; 2019. Accessed April 23, 2024. https://www.msdvetmanual.com/all‐other‐pets/guinea‐pigs/providing‐a‐home‐for‐a‐guinea‐pig

[17] Neville V , Mounty J , Benato L , Hunter K , Mendl M , Paul ES . Pet rat welfare in the United Kingdom: the good, the bad and the ugly. Vet Rec. 2021;189:e559. 34101201 10.1002/vetr.559

[18] Davies A , Adler A , Leonard D , Allcock J , Curwen A . Shortage of experienced vets. Vet Rec. 2015;177:447. 10.1136/vr.h5755 26515353

[19] Wills A , Holt S . Confidence of veterinary surgeons in the United Kingdom in treating and diagnosing exotic pet species. Vet Rec. 2020;186:e20. 10.1136/vr.105664 PMC7365564 32015163

[20] Hedley JE , Pettitt A , Abeyesinghe SM . Preliminary investigation into the housing of dwarf hamsters. Vet Rec. 2023;193:e3170. 37527402 10.1002/vetr.3170

[21] Williams A , Williams B , Hansen CR , Coble KH . The impact of pet health insurance on dog owners’ spending for veterinary services. Animals. 2020;10:1162. 32659934 10.3390/ani10071162 PMC7401533

[22] PDSA . PDSA animal wellbeing report 2023. Accessed April 23, 2024. https://www.pdsa.org.uk/what‐we‐do/pdsa‐animal‐wellbeing‐report/paw‐report‐2023

[23] Springer S , Lund TB , Grimm H , Kristensen AT , Corr SA , Sandøe P . Comparing veterinarians’ attitudes to and the potential influence of pet health insurance in Austria, Denmark and the UK. Vet Rec. 2022;190:e1266. 34997603 10.1002/vetr.1266

[24] Rooney NJ , Blackwell EJ , Mullan SM , Saunders R , Baker PE , Hill JM , et al. The current state of welfare, housing and husbandry of the English pet rabbit population. BMC Res Notes. 2014;7:942. 25532711 10.1186/1756-0500-7-942 PMC4307134

[25] Westgarth C , Heron J , Ness AR , Bundred P , Gaskell RM , Coyne KP , et al. Family pet ownership during childhood: Findings from a UK birth cohort and implications for public health research. Int J Environ Res Public Health. 2010;7:3704–3729. 21139856 10.3390/ijerph7103704 PMC2996187

[26] Edgar JL , Mullan SM . Knowledge and attitudes of 52 UK pet rabbit owners at the point of sale. Vet Rec. 2011;168:353. 21498237 10.1136/vr.c6191

[27] Turner WG . Our new children: the surrogate role of companion animals in women's lives. Qual Rep. 2001;6:1–10.

[28] Mueller MK , King EK , Callina K , Dowling‐Guyer S , McCobb E . Demographic and contextual factors as moderators of the relationship between pet ownership and health. Heal Psychol Behav Med. 2021;9:701–723. 10.1080/21642850.2021.1963254 PMC8356805 34395058

[29] Roberts‐Steel S , Oxley JA , Carroll A , Wills AP . Frequency of owner‐reported bacterial infections in pet guinea pigs. Animals. 2019;9:649. 31487781 10.3390/ani9090649 PMC6770499

[30] Rioja‐Lang F , Bacon H , Connor M , Dwyer CM . Rabbit welfare: determining priority welfare issues for pet rabbits using a modified Delphi method. Vet Rec Open. 2019;6:e000363. 31903189 10.1136/vetreco-2019-000363 PMC6924855

[31] Norman R , Wills AP . An investigation into the relationship between owner knowledge, diet, and dental disease in guinea pigs (*Cavia porcellus*). Animals. 2016;6:73. 27854241 10.3390/ani6110073 PMC5126775

[32] Gattermann R , Johnston RE , Yigit N , Fritzsche P , Larimer S , Ozkurt S , et al. Golden hamsters are nocturnal in captivity but diurnal in nature. Biol Lett. 2008;4:253–255. 18397863 10.1098/rsbl.2008.0066 PMC2610053

[33] Simasko SM , Mukherjee S . Novel analysis of sleep patterns in rats separates periods of vigilance cycling from long‐duration wake events. Behav Brain Res. 2009;196:228–236. 18835301 10.1016/j.bbr.2008.09.003 PMC2617706

[34] Welch T , Coe JB , Niel L , McCobb E . A survey exploring factors associated with 2890 companion‐rabbit owners’ knowledge of rabbit care and the neuter status of their companion rabbit. Prev Vet Med. 2017;137:13–23. 28107876 10.1016/j.prevetmed.2016.12.008

[35] Weiss E , Dolan ED , Garrison L , Hong J , Slater M . Should dogs and cats be given as gifts? Animals. 2013;3(4):995–1001. 26479748 10.3390/ani3040995 PMC4494363

[36] Smith KM , Smith KF , D'Auria JP . Exotic pets: health and safety issues for children and parents. J Pediatr Heal Care. 2012;26:e2–e6. 10.1016/j.pedhc.2011.11.009 22206642

[37] Rowland M . Veterinary care of guinea pigs. In Pract. 2020;42:91–104.

[38] Anderson KL , Casey RA , Cooper B , Upjohn MM , Christley RM . National dog survey: describing UK dog and ownership demographics. Animals. 2023;13:1072. 36978614 10.3390/ani13061072 PMC10044414

[39] Kipperman BS , Kass PH , Rishniw M . Factors that influence small animal veterinarians’ opinions and actions regarding cost of care and effects of economic limitations on patient care and outcome and professional career satisfaction and burnout. J Am Vet Med Assoc. 2017;250:785–794. 28306486 10.2460/javma.250.7.785

[40] Clark D . Average annual disposable income in the UK 2021/22, by age group. Statistica; 2024. Accessed May 1, 2024. https://www.statista.com/statistics/824464

[41] Gerrard E . Owner compliance—educating clients to act on pet care advice. VN Times. 2015;15:6–7.

[42] Rollin B . The ethics of referral. Can Vet J. 2006;47:717. 16898119 PMC1482440

[43] LaVallee E , Mueller MK , McCobb E . A systematic review of the literature addressing veterinary care for underserved communities. J Appl Anim Welf Sci. 2017;20:381–394. 28657796 10.1080/10888705.2017.1337515

[44] Lupu G , Bel L , Andrei S . Pain management and analgesics used in small mammals during post‐operative period with an emphasis on metamizole (dipyrone) as an alternative medication. Molecules. 2022;27:7434. 36364259 10.3390/molecules27217434 PMC9657641

[45] Williams A , Coble KH , Williams B , Dicks M , Knippenberg R . Consumer preferences for pet health insurance. Annual Meeting; 6–9 February 2016, San Antonio, TX. Southern Agricultural Economics Association; 2016. p. 230144.

[46] Sánchez‐Vizcaíno F , Noble P‐JM , Jones PH , Menacere T , Buchan I , Reynolds S , et al. Demographics of dogs, cats, and rabbits attending veterinary practices in Great Britain as recorded in their electronic health records. BMC Vet Res. 2017;13:218. 28693574 10.1186/s12917-017-1138-9 PMC5504643

[47] Brockman BK , Taylor VA , Brockman CM . The price of unconditional love: consumer decision making for high‐dollar veterinary care. J Bus Res. 2008;61:397–405.

[48] Riggs SM . Guinea pigs. In: Manual of exotic pet practice. Elsevier Health Sciences; 2008. p. 456–473.

[49] King CA , Smith TJ , Holman E , Serpell JA , Grandin T . Medical, behavioral and abuse status characteristics: predictors of perceived adoptability, appeal, and resource demands of shelter dogs. Anthrozoos. 2021;34:507–524.

[50] Hou CY , Protopopova A . Rats as pets: predictors of adoption and surrender of pet rats (*Rattus norvegicus domestica*) in British Columbia, Canada. PLoS One. 2022;17:e0264262. 35180270 10.1371/journal.pone.0264262 PMC8856535

[51] Cleland JA . The qualitative orientation in medical education research. Korean J Med Educ. 2017;29:61. 28597869 10.3946/kjme.2017.53 PMC5465434

[52] Coe JB , Adams CL , Bonnett BN . A focus group study of veterinarians’ and pet owners’ perceptions of the monetary aspects of veterinary care. J Am Vet Med Assoc. 2007;231:1510–1518. 18020992 10.2460/javma.231.10.1510

