# Supplementary material for: Perception and utilisation of veterinary services by rodent owners in the United Kingdom
Source: Vet Rec. 2025 Jan 25;196(8):e4958. doi: 10.1002/vetr.4958 (PMC12007489; doi:10.1002/vetr.4958)
Supplement: Supplementary file 1 — Supporting Information [file VETR-196-e4958-s001.pdf]

Supplementary Material 1. Pairwise comparisons of the effect of annual income, species owned and where the pet was obtained on owner perceived confidence in assessing when their pet is ill. *<0.05 **<0.001. Mean ± standard deviation are shown underneath each category group. Original answers were coded based on a Likert-scale. 1=Strongly Agree, 2=Agree, 3=Neither agree nor disagree, 4=Disagree, 5=Strongly Disagree.

**Effect of Income on 'I am confident in assessing when my pet is ill'**	
Owner Income	p-value
Below £10,000 (1.65 ± 0.709) and £10,001 - £20,000 (1.58 ± 0.737)	1.000
Below £10,000 (1.65 ± 0.709) and £20,001 - £30,000 (1.65 ± 0.772)	1.000
Below £10,000 (1.65 ± 0.709) and £30,001- £40,000 (1.84 ± 0.863)	0.259
Below £10,000 (1.65 ± 0.709) and £40,001 - £50,000 (1.89 ± 0.769)	0.039*
Below £10,000 (1.65 ± 0.709) and above £50,000 (1.88 ± 0.829)	0.106
£10,001-£20,000 (1.58 ± 0.737) and £20,001 - £30,000 (1.65 ± 0.772)	1.000
£10,001-£20,000 (1.58 ± 0.737) and £30,001- £40,000 (1.84 ± 0.863)	0.127
£10,001-£20,000 (1.58 ± 0.737) and £40,001 - £50,000 (1.89 ± 0.769)	0.020*
£10,001-£20,000 (1.58 ± 0.737) and above £50,000 (1.88 ± 0.829)	0.056
£20,001-£30,000 (1.65 ± 0.772) and £30,001- £40,000 (1.84 ± 0.863)	0.040*
£20,001-£30,000 (1.65 ± 0.772) and £40,001 - £50,000 (1.89 ± 0.769)	0.007*
£20,001-£30,000 (1.65 ± 0.772) and above £50,000 (1.88 ± 0.829)	0.021*
£30,001-£40,000 (1.84 ± 0.863) and £40,001 - £50,000 (1.89 ± 0.769)	1.000
£30,001-£40,000 (1.84 ± 0.863) above £50,000 (1.88 ± 0.829)	1.000
£40,001 - £50,000 (1.89 ± 0.769) and above £50,000 (1.88 ± 0.829)	1.000
**Effect of Species Owned on 'I am confident in assessing when my pet is ill'**	
Species Owned	p-value
Guinea Pig (1.70 ± 0.788) and Gerbil (1.74 ± 0.754)	1.000
Guinea Pig (1.70 ± 0.788) and Rat (1.51 ± 0.652)	0.001*
Guinea Pig (1.70 ± 0.788) and Hamster (1.77 ± 0.795)	1.000
Guinea Pig (1.70 ± 0.788) and Mouse (1.72 ± 0.809)	1.000
Gerbil (1.74 ± 0.754) and Rat (1.51 ± 0.652)	0.123
Gerbil (1.74 ± 0.754) and Hamster (1.77 ± 0.795)	1.000
Gerbil (1.74 ± 0.754) and Mouse (1.72 ± 0.809)	1.000

Rat (1.51 ± 0.652) and Hamster (1.77 ± 0.795)	<0.001**
Rat (1.51 ± 0.652) and Mouse (1.72 ± 0.809)	0.958
Hamster (1.77 ± 0.795) and Mouse (1.72 ± 0.809)	1.000
**Effect of Where Pet was Obtained on 'I am confident in assessing when my pet is ill'**	
Where Pet was Obtained	p-value
Pet Shop (1.80 ± 0.813) and Rescue Centre (1.62 ± 0.722)	0.002*
Pet Shop (1.80 ± 0.813) and Breeder (1.59 ± 0.703)	<0.001**
Pet Shop (1.80 ± 0.813) and Gifted (2.06 ± 0.736)	0.213
Pet Shop (1.80 ± 0.813) and Other (1.74 ± 0.828)	1.000
Rescue Centre (1.62 ± 0.722) and Breeder (1.59 ± 0.703)	1.000
Rescue Centre (1.62 ± 0.722) and Gifted (2.06 ± 0.736)	0.003*
Rescue Centre (1.62 ± 0.722) and Other (1.74 ± 0.828)	1.000
Breeder (1.59 ± 0.703) and Gifted (2.06 ± 0.736)	0.002*
Breeder (1.59 ± 0.703) and Other (1.74 ± 0.828)	0.754
Gifted (2.06 ± 0.736) and Other (1.74 ± 0.828)	0.061
