# Supplementary material for: Perception and utilisation of veterinary services by rodent owners in the United Kingdom
Source: Vet Rec. 2025 Jan 25;196(8):e4958. doi: 10.1002/vetr.4958 (PMC12007489; doi:10.1002/vetr.4958)
Supplement: Supplementary file 2 — Supporting Information [file VETR-196-e4958-s003.pdf]

Supplementary Material 2. Pairwise comparisons of the effect of age group, species owned and where the pet was obtained on owner willingness to pay for medication for their rodent. *<0.05 **<0.001. Mean ± standard deviation are located underneath each category group. Original answers were coded based on a Likert-scale. 1=Strongly Agree, 2=Agree, 3=Neither agree nor disagree, 4=Disagree, 5=Strongly Disagree

**Effect of age on 'I would be willing to pay for medicine for my pet'**	
Owner Age	p-value
18-24 (1.17 ± 0.423) and 25-34 (1.18 ± 0.442)	0.037*
18-24 (1.17 ± 0.423) and 35-44 (1.30 ± 0.545)	<0.001**
18-24 (1.17 ± 0.423) and 45-54 (1.32 ± 0.507)	<0.001**
18-24 (1.17 ± 0.423) and 55-64 (1.25 ± 0.468)	0.020*
18-24 (1.17 ± 0.423) and 65+ (1.14 ± 0.359)	0.065
25-34 (1.18 ± 0.442) and 35-44 (1.30 ± 0.545)	0.340
25-34 (1.18 ± 0.442) and 45-54 (1.32 ± 0.507)	0.024*
25-34 (1.18 ± 0.442) and 55-64 (1.25 ± 0.468)	1.000
25-34 (1.18 ± 0.442) and 65+ (1.14 ± 0.359)	0.696
35-44 (1.30 ± 0.545) and 45-54 (1.32 ± 0.507)	1.000
35-44 (1.30 ± 0.545) and 55-64 (1.25 ± 0.468)	1.000
35-44 (1.30 ± 0.545) and 65+ (1.14 ± 0.359)	1.000
45-54 (1.32 ± 0.507) and 55-64 (1.25 ± 0.468)	1.000
45-54 (1.32 ± 0.507) and 65+ (1.14 ± 0.359)	1.000
**Effect of Species Owned on 'I would be willing to pay for medicine for my pet'**	
Species Owned	p-value
Guinea Pig (1.24 ± 0.464) and Gerbil (1.30 ± 0.593)	1.000
Guinea Pig (1.24 ± 0.464) and Rat (1.12 ± 0.348)	0.001*
Guinea Pig (1.24 ± 0.464) and Hamster (1.24 ± 0.494)	1.000
Guinea Pig (1.24 ± 0.464) and Mouse (1.36 ± 0.722)	1.000
Gerbil (1.30 ± 0.593) and Rat (1.12 ± 0.348)	0.074
Gerbil (1.30 ± 0.593) and Hamster (1.24 ± 0.494)	1.000
Gerbil (1.30 ± 0.593) and Mouse (1.36 ± 0.722)	1.000

Rat (1.12 ± 0.348) and Hamster (1.24 ± 0.494)	0.007*
Rat (1.12 ± 0.348) and Mouse (1.36 ± 0.722)	0.128
Hamster (1.24 ± 0.494) and Mouse (1.36 ± 0.722)	1.000
**Effect of Where Pet was Obtained on ‘I would be willing to pay for medicine for my pet’**	
Where Pet was Obtained	p-value
Pet Shop (1.30 ± 0.551) and Rescue Centre (1.17 ± 0.392)	<0.001**
Pet Shop (1.30 ± 0.551) and Breeder (1.19 ± 0.431)	0.009*
Pet Shop (1.30 ± 0.551) and Gifted (1.29 ± 0.462)	1.000
Pet Shop (1.30 ± 0.551) and Other (1.17 ± 0.429)	0.007*
Rescue Centre (1.17 ± 0.392) and Breeder (1.19 ± 0.431)	1.000
Rescue Centre (1.17 ± 0.392) Gifted (1.29 ± 0.462)	0.717
Rescue Centre (1.17 ± 0.392) and Other (1.17 ± 0.429)	1.000
