# Supplementary figures and images for: Perception and utilisation of veterinary services by rodent owners in the United Kingdom
Source: Vet Rec. 2025 Jan 25;196(8):e4958. doi: 10.1002/vetr.4958 (PMC12007489; doi:10.1002/vetr.4958)

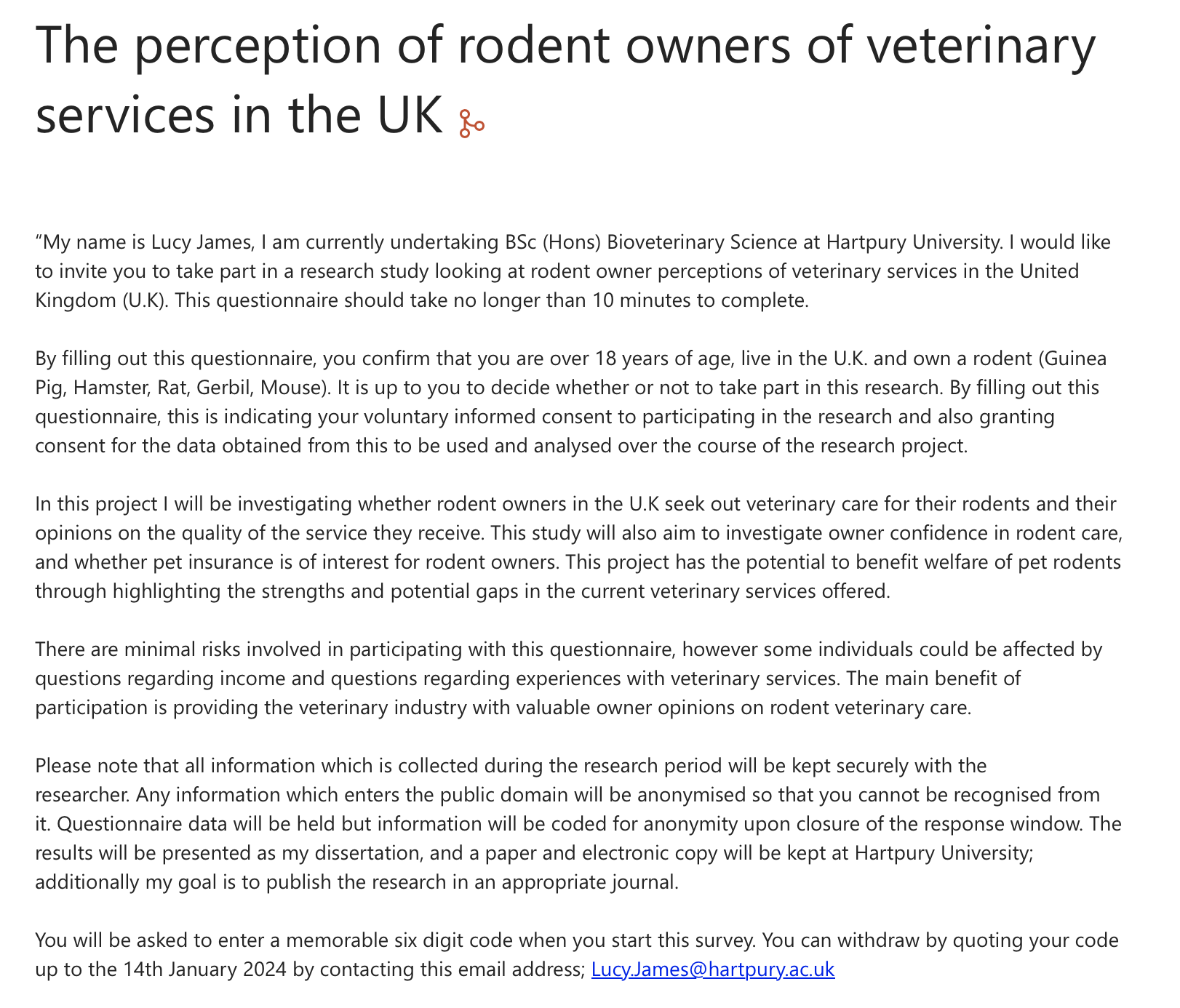


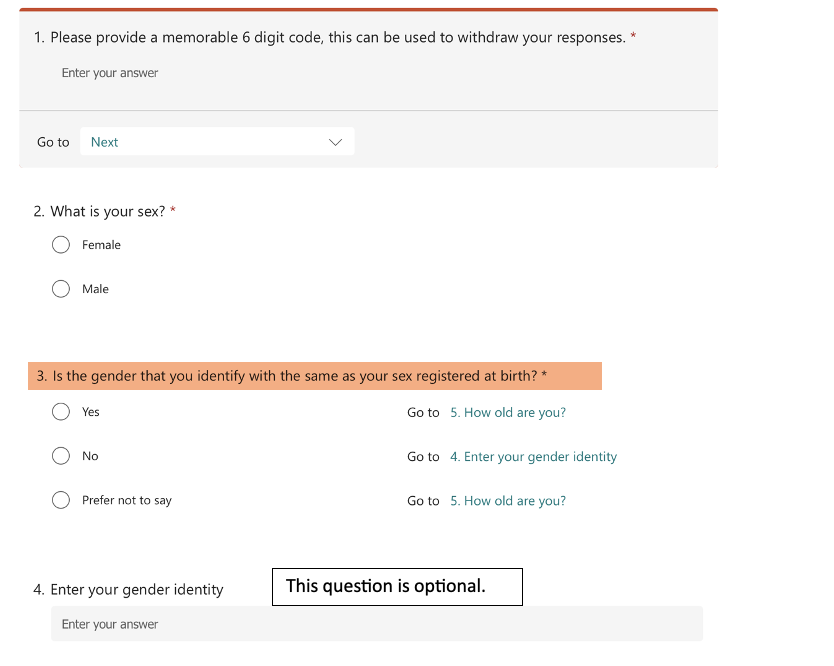


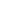


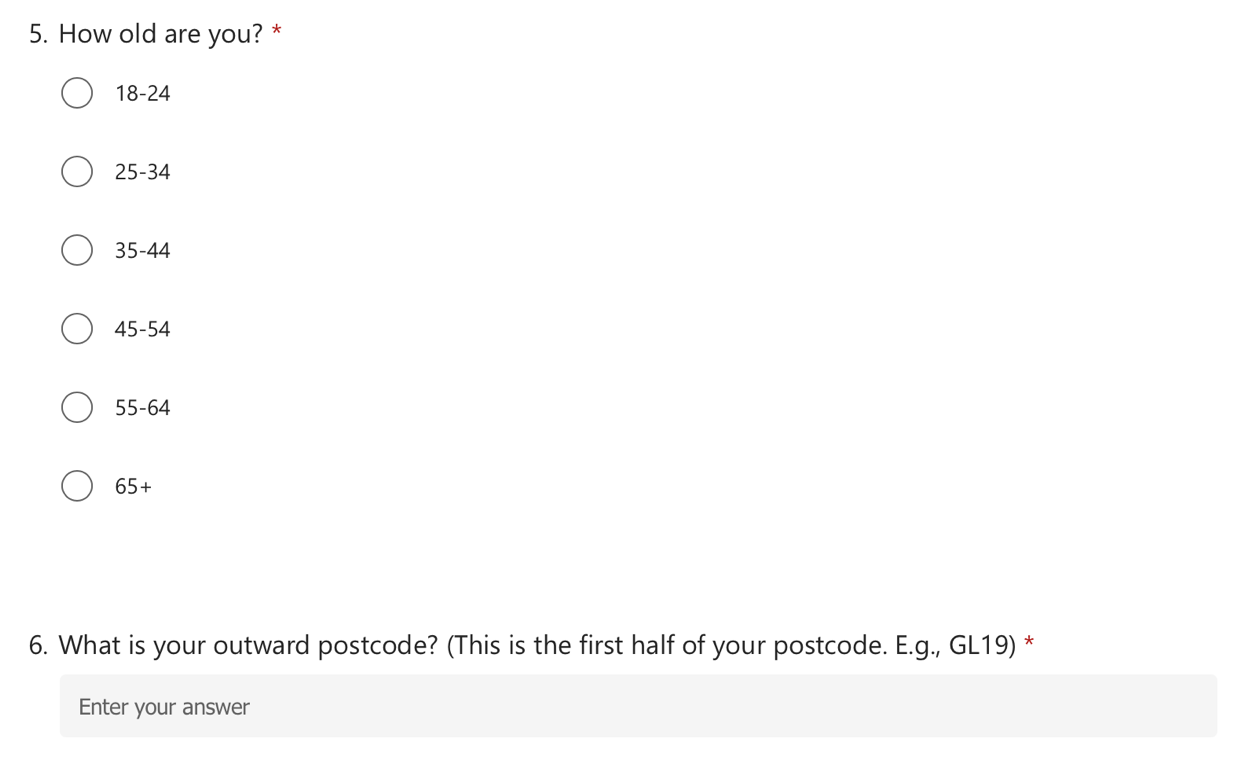


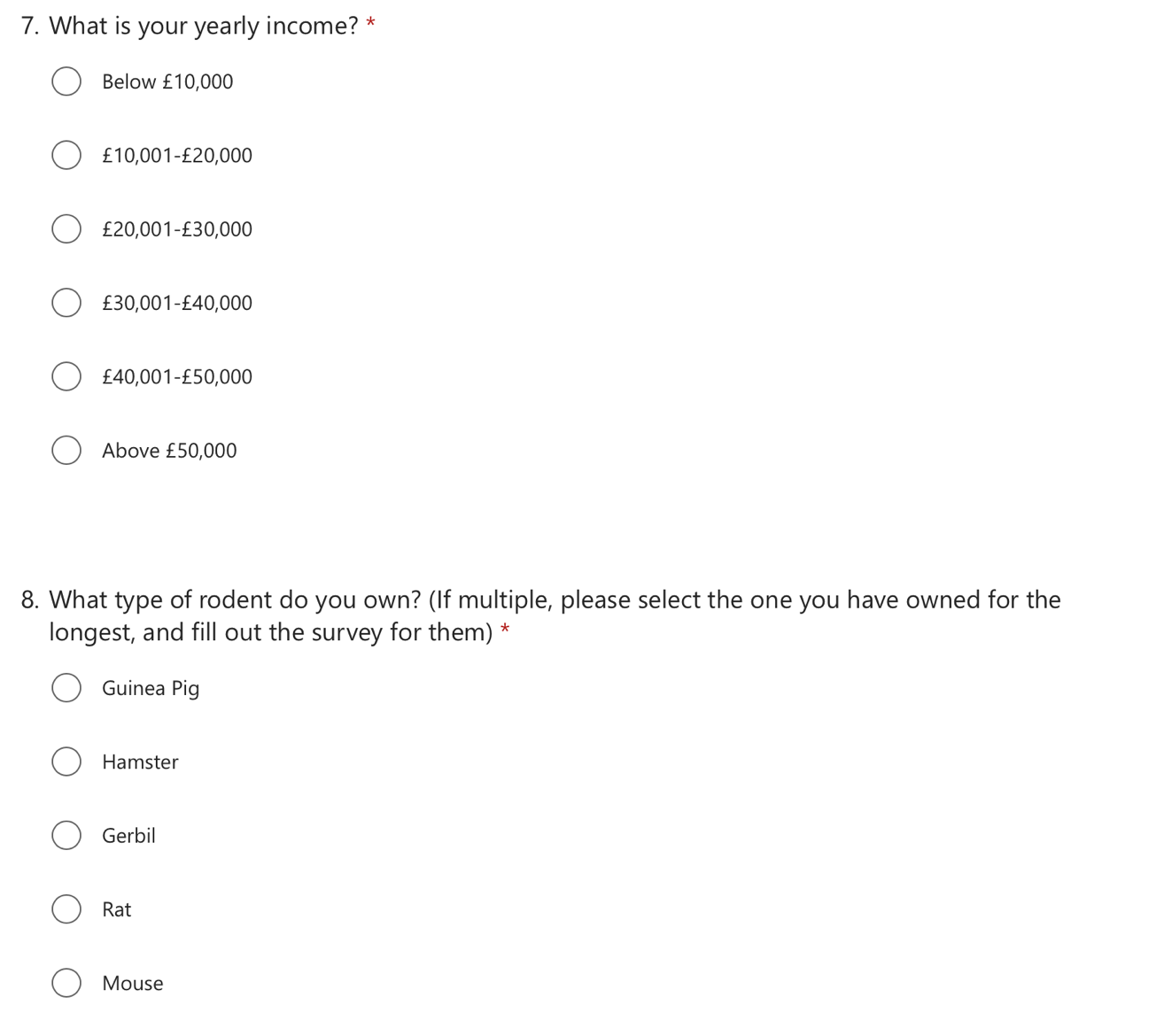


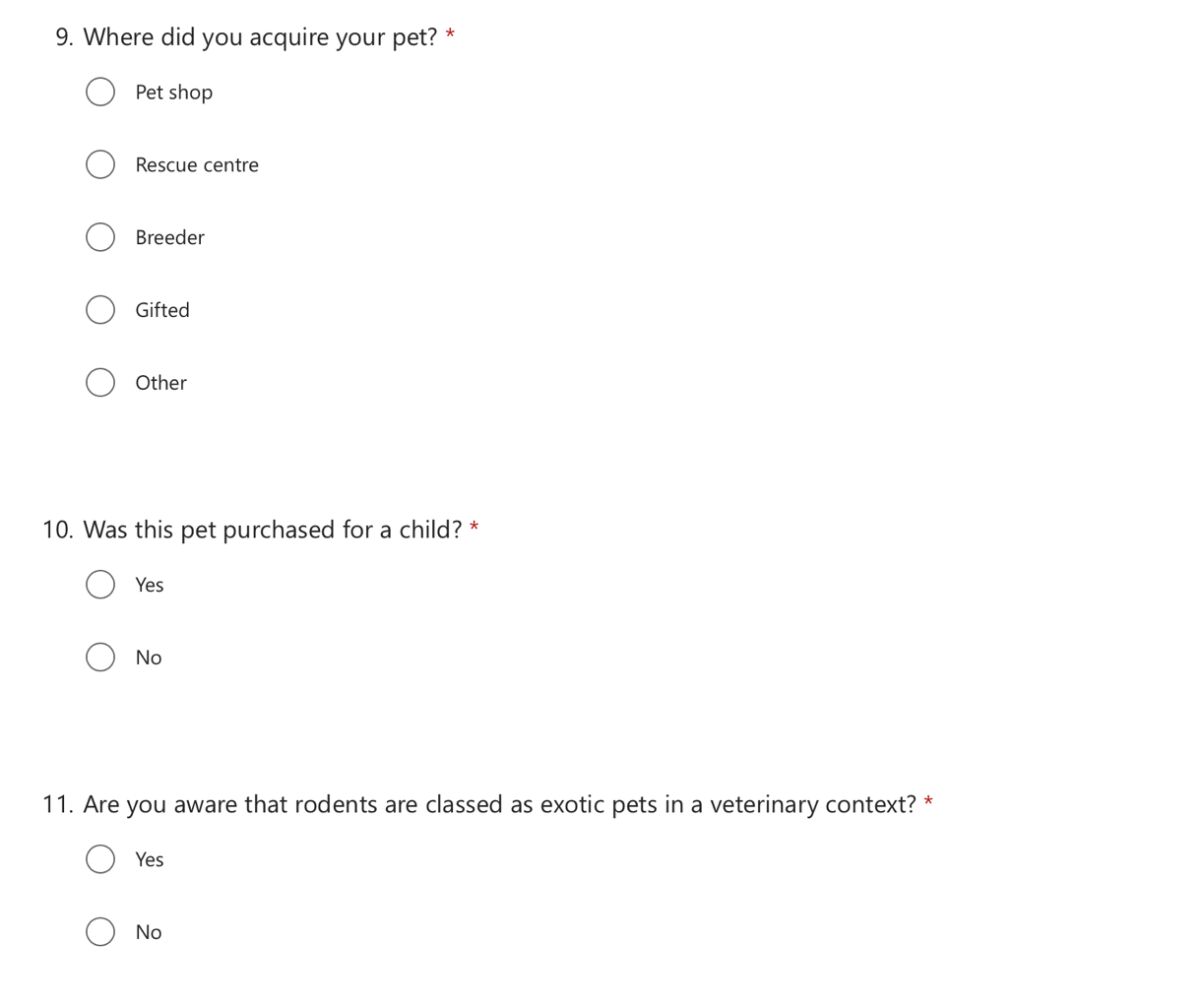


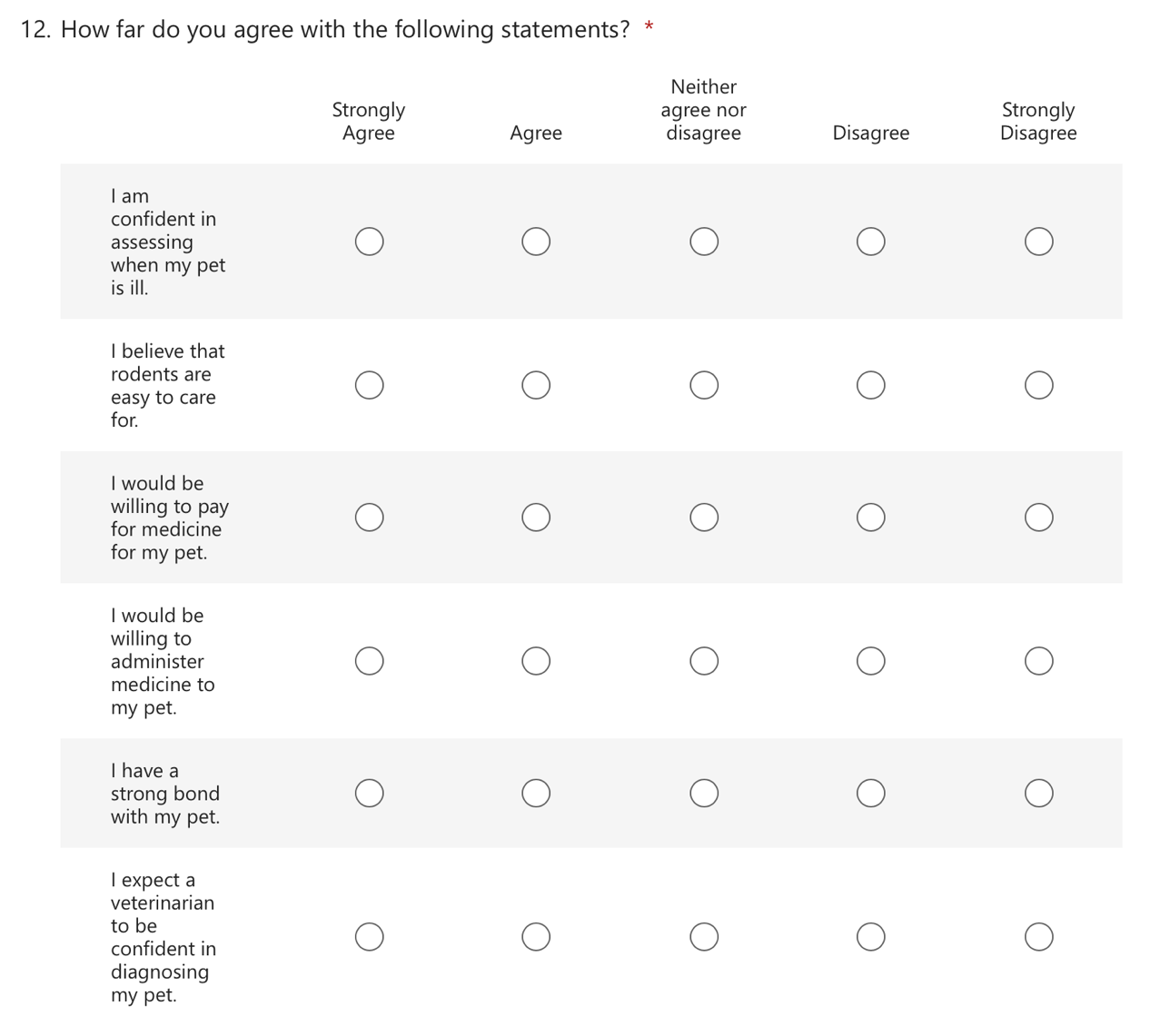

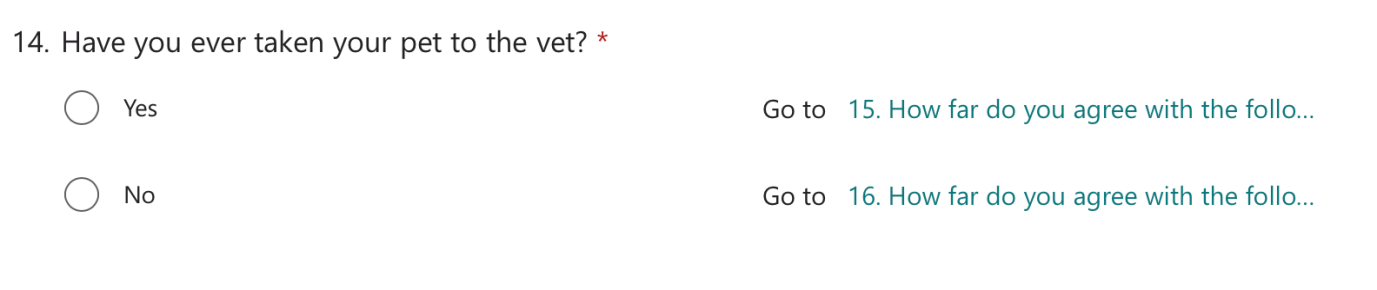

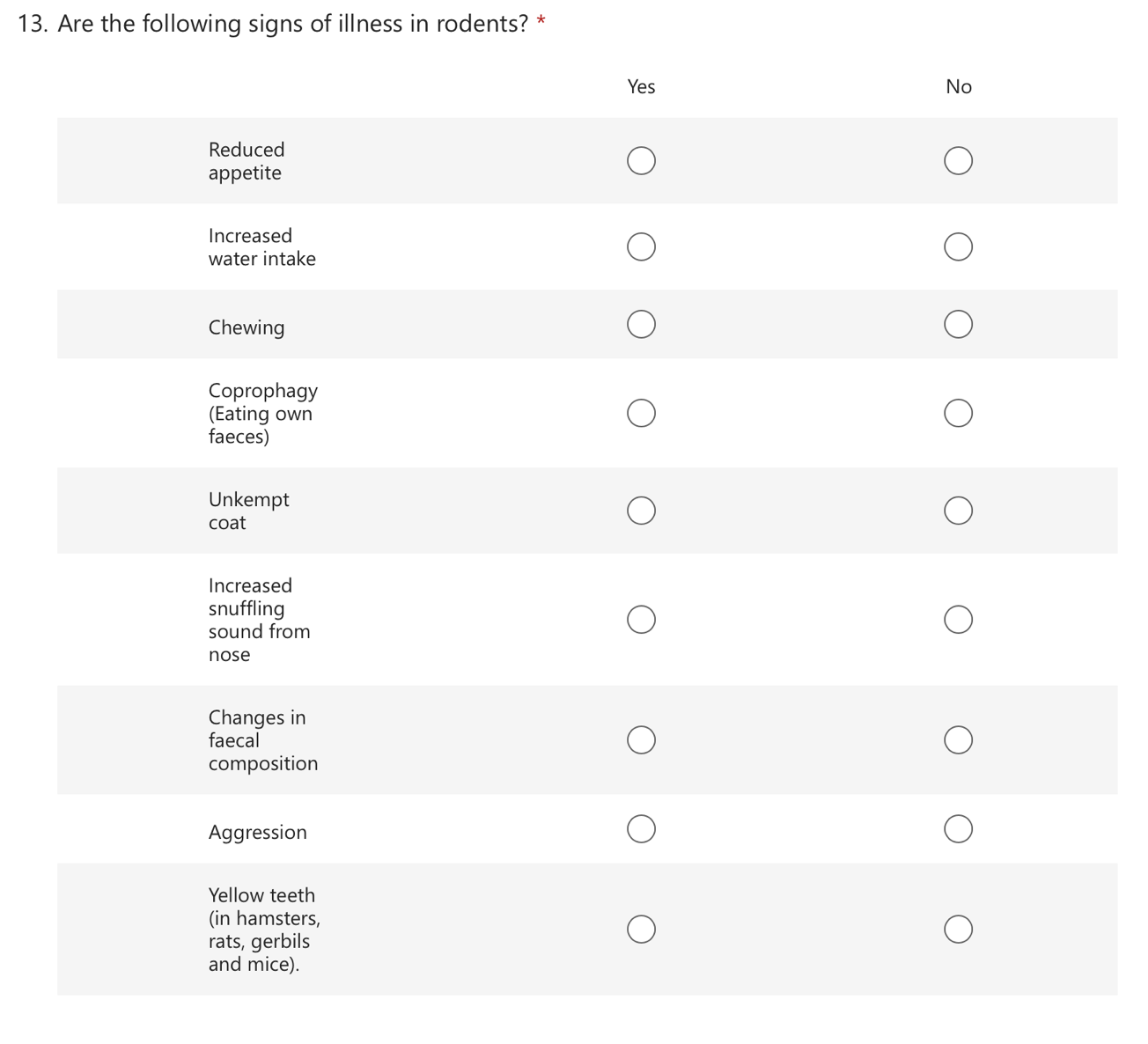


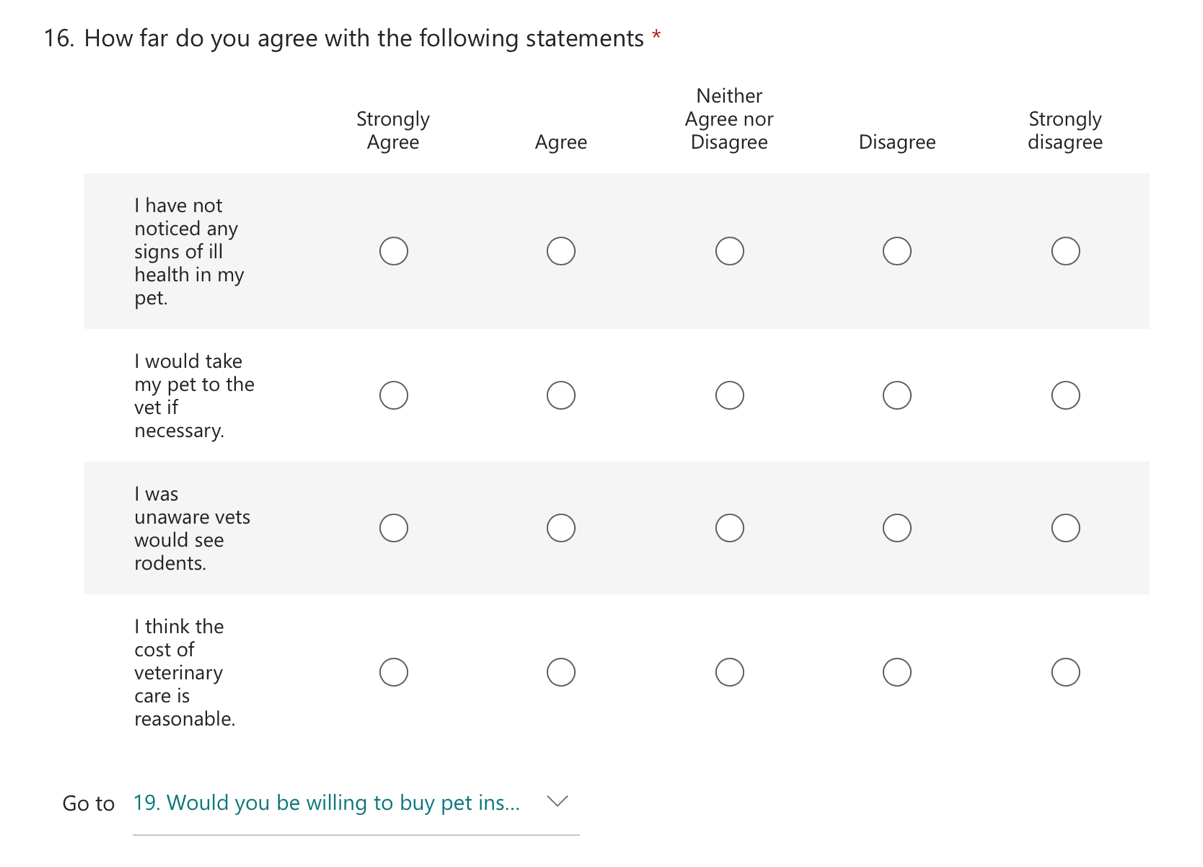

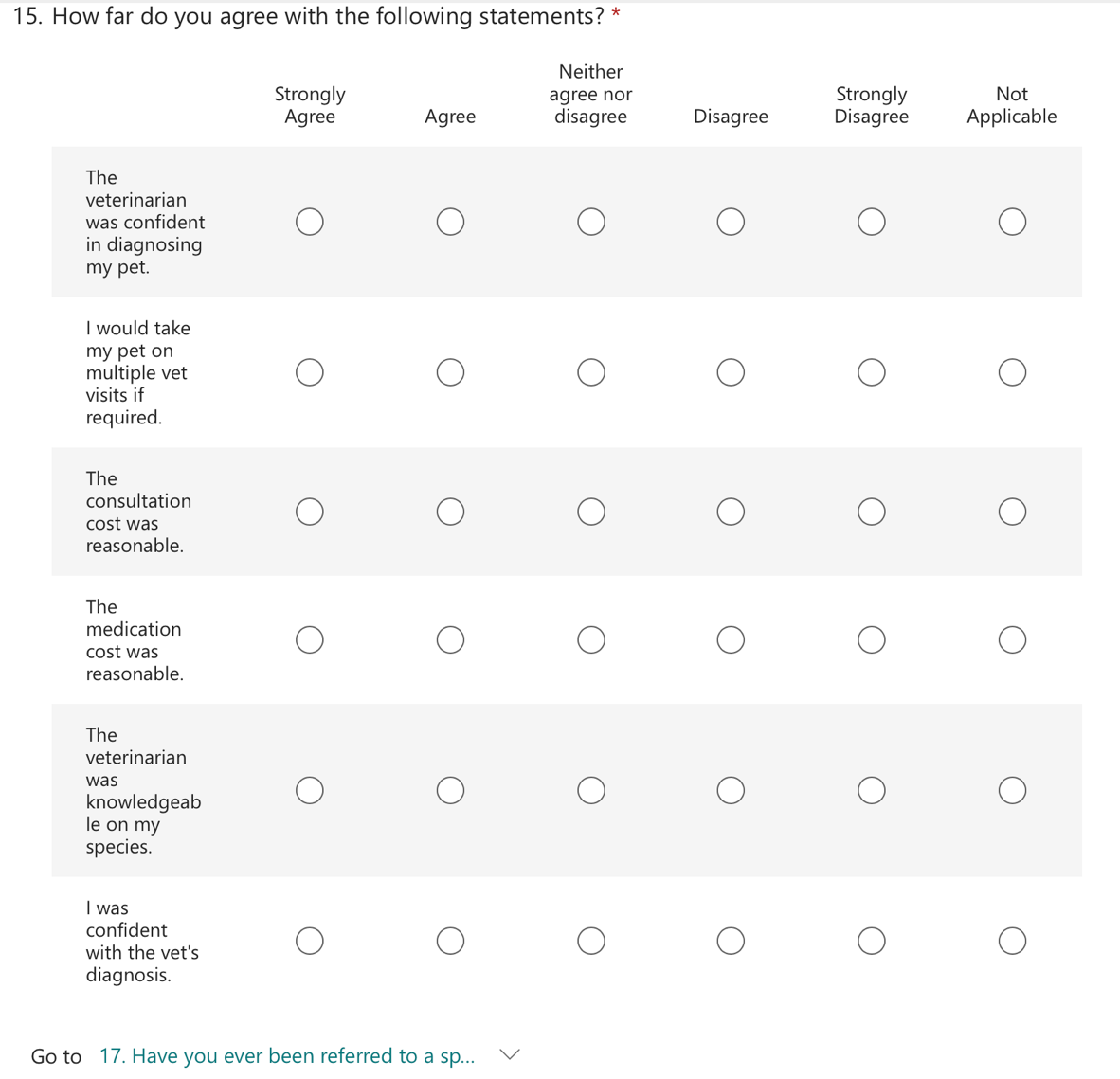


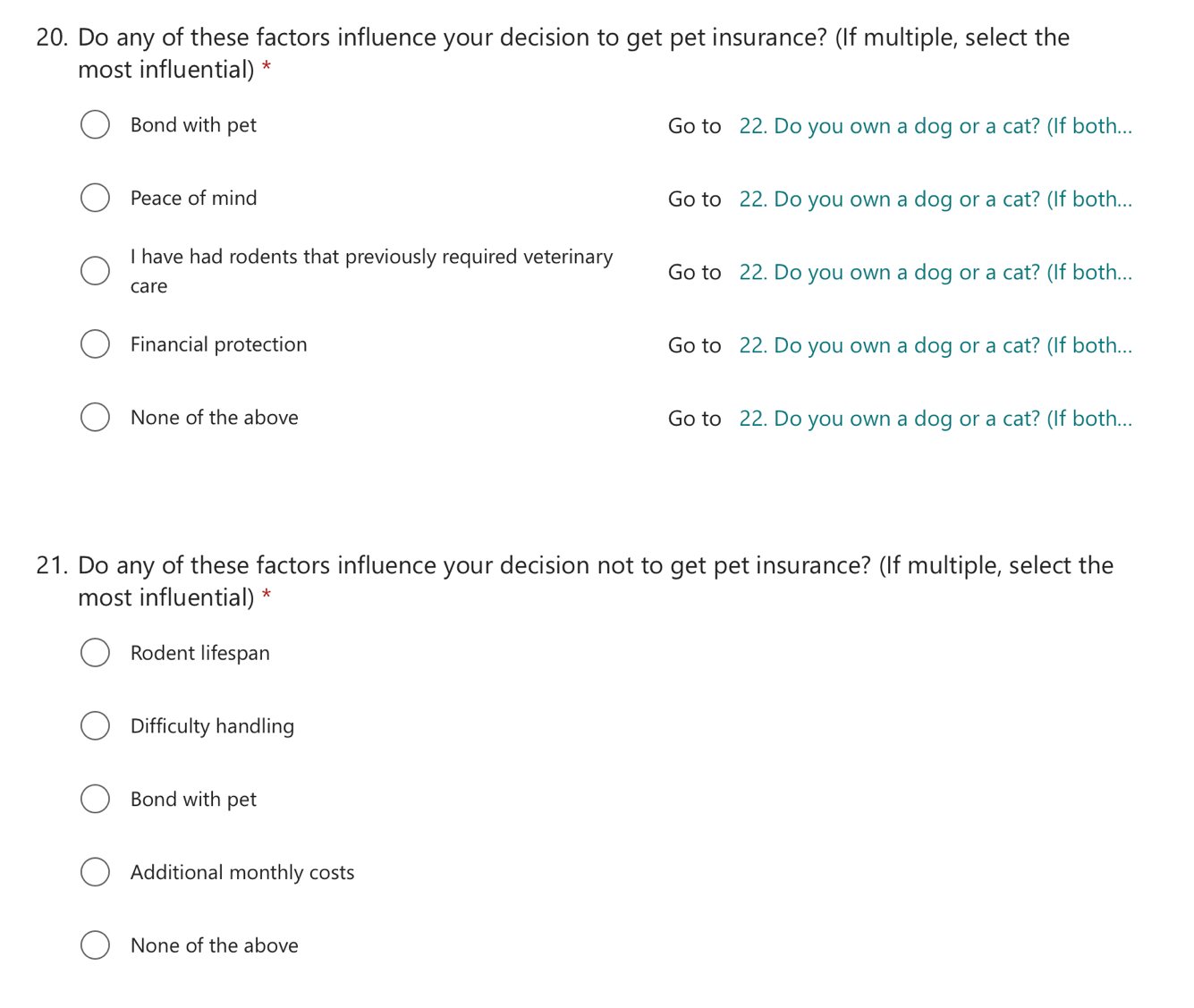

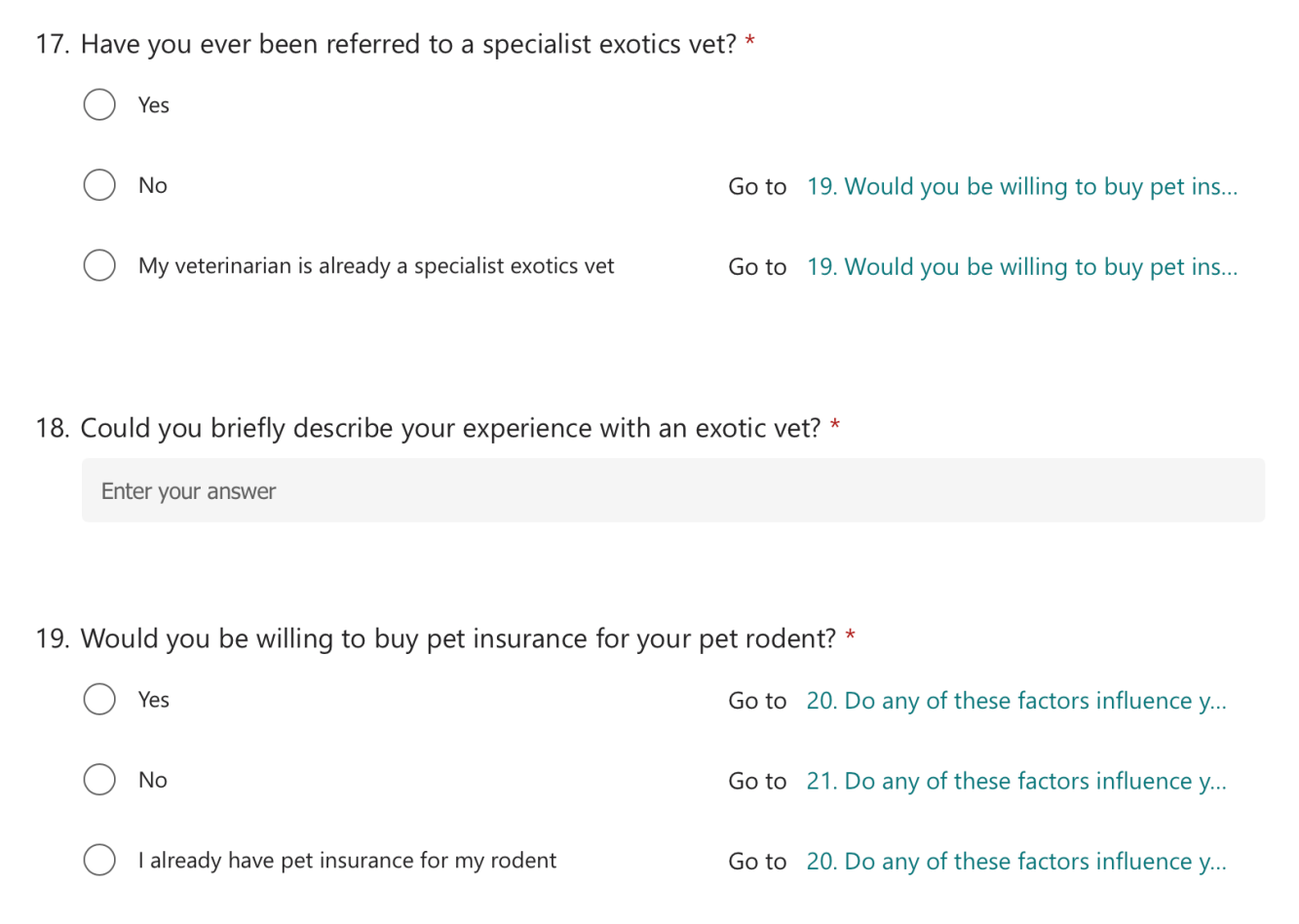


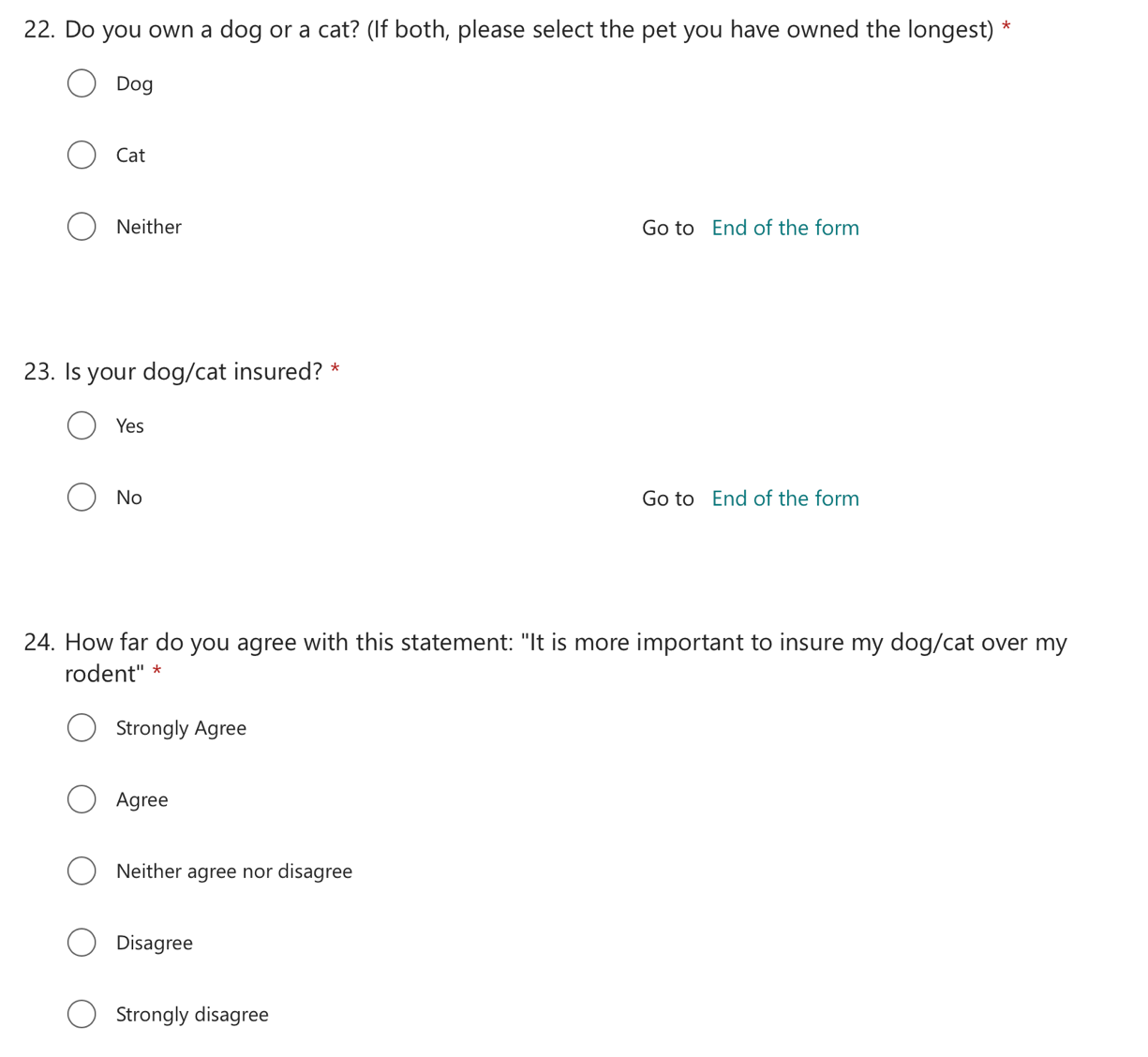


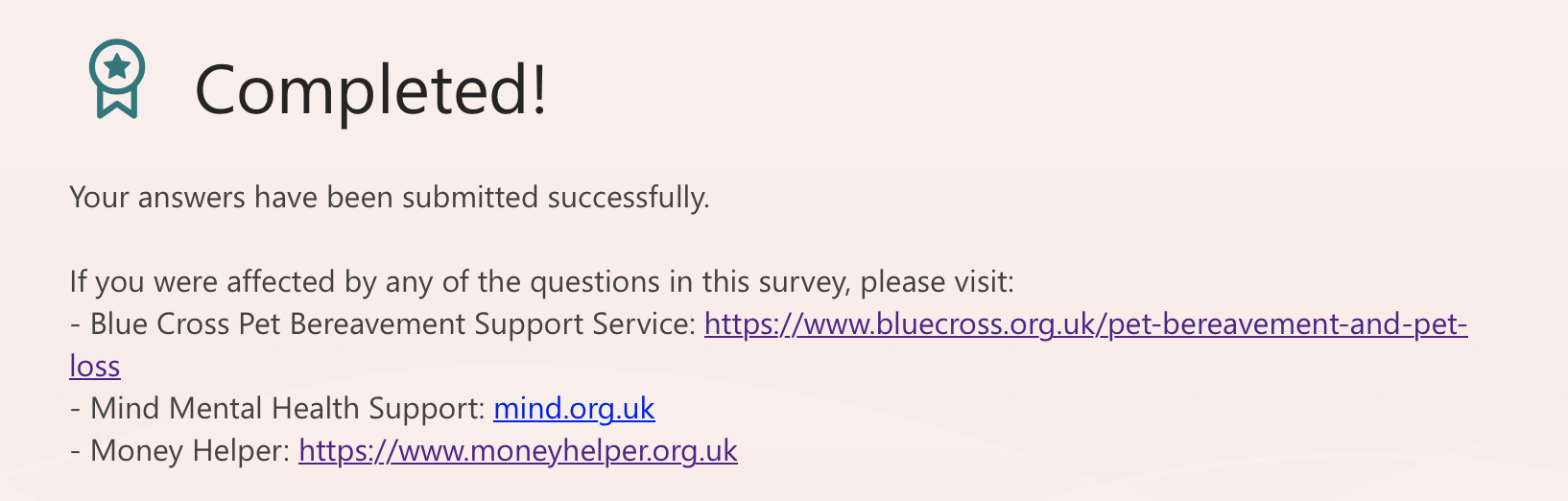

Supplement: Supplementary file 3 — Supporting Information [file VETR-196-e4958-s002.docx]
